# Draft Genome Sequence of *Acinetobacter* sp. Strain VT-511 Isolated from the Stomach of a Patient with Gastric Cancer

**DOI:** 10.1128/genomeA.01202-15

**Published:** 2015-10-15

**Authors:** George Tetz, Victor Tetz

**Affiliations:** a Institute of Human Microbiology, New York, New York, USA; b First State I. P. Pavlov Medical University, Saint Petersburg, Russia

## Abstract

We report the draft genome sequence of *Acinetobacter* sp. strain VT-511, which was obtained from the stomach of a patient with gastric cancer. The genome of *Acinetobacter* sp. VT-511 is composed of approximately 3,416,321 bp and includes 3,214 predicted protein-coding genes.

## GENOME ANNOUNCEMENT

The genus *Acinetobacter* includes aerobic, nonmotile, Gram-negative coccobacilli. Species of this genus are widely distributed in nature and have been isolated from soil and water (1). *Acinetobacter* spp. are associated with nosocomial infections, including pneumonia, meningitis, and sepsis (2-4). In this study, we sequenced and analyzed the genome of clinically isolated *Acinetobacter* sp. strain VT-511 that was isolated from the stomach of a patient with gastric cancer. 

Comparative 16S rRNA analysis revealed 99% sequence identity with *Acinetobacter schindleri* NIPH 900, a bacterium found on human conjunctiva and not associated with gut microbiota (5).

*Acinetobacter* sp. VT-511 was then characterized using biochemical analysis and matrix-assisted laser desorption ionization–time of flight (MALDI-TOF) mass spectrometry that revealed differences with other *A. schindleri* strains.

Whole-genome sequencing was performed using the Illumina MiSeq HiSeq 2500 according to the manufacturer’s instructions (Illumina, Inc., CA). The generated reads were trimmed and assembled *de novo* with SPAdes genome assembler software, version 3.5.0 (6). A total of 121 contigs with an average coverage of 125× were generated. The draft genome of *Acinetobacter* sp. VT-511 consisted of 3,416,321 bp and had a G+C content of 42.7%.

Sequence annotation was performed using the NCBI Prokaryotic Genome Annotation Pipeline and by using RAST (7, 8). The genome of *Acinetobacter* sp. VT-511 was composed of 3,214 predicted protein-coding sequences and 96 predicted RNA genes, including 14 rRNAs, 81 tRNAs, and 1 noncoding RNA. The analysis revealed that the predicted gene sequences for multidrug resistance transporters of ABC, MATE, and RND families and macrolide-specific efflux proteins were also present. In addition, genes encoding resistance to ethidium bromide, fluoroquinolones, macrolides, fosmidomycin, and bacteriocin were identified. The genome also harbored genes encoding virulence factors such as adhesins, polysaccharides, peptidases, and proteases (9, 10).

The genome size of *Acinetobacter* sp. VT-511 (3,416,321 bp) was larger than that of *A. schindleri* NIPH 900 (3,404,540 bp), which is the phylogenetically closest organism. However, the number of protein-coding genes harbored by *Acinetobacter* sp. VT-511 (3,214 genes) was lower than the number harbored by *A. schindleri* NIPH 900 (3,232 genes). The G+C contents of *Acinetobacter* sp. VT-511 and *A. schindleri* NIPH 900 were 42.7% and 42.2%, respectively.

Digital DNA-DNA hybridization (DDH) was performed using *Acinetobacter* sp. VT-511 and *A. schindleri* NIPH 900 and assessed with GGDC 2.0. The obtained DDH value of 75.6% was below the threshold value of 79% set for genomes belonging to the same subspecies (11, 12). *Acinetobacter* sp. VT-511 harbored genes involved in the following processes: metabolism of aromatic compounds, capsular and extracellular polysaccharide expression, antibiotic resistance, and the YefM-YoeB toxin antitoxin system. These genes were not present in *A. schindleri* NIPH 900. The regions encoding genes involved in biotin synthesis, transcriptional regulators, oxidative stress response, and recycling of peptidoglycan had sequence similarities lower than 50% between the two strains.

In conclusion, the draft genome sequence of *Acinetobacter* sp. VT-511 was determined. The findings of this study may be useful for identification of *Acinetobacter* species in microbiota of patients with gastrointestinal tract malignancies.

### Nucleotide sequence accession number.

This genome sequencing project has been deposited in GenBank under the accession no. LFRE00000000.
